# *Castanea sativa Mill.* bark extract exhibits chemopreventive properties triggering extrinsic apoptotic pathway in Jurkat cells

**DOI:** 10.1186/s12906-017-1756-6

**Published:** 2017-05-05

**Authors:** Monia Lenzi, Marco Malaguti, Veronica Cocchi, Silvana Hrelia, Patrizia Hrelia

**Affiliations:** 1 Department of Pharmacy and Biotechnology, University of Bologna, Via San Donato 15, 40127 Bologna, Italy; 2 Department for Life Quality Studies, University of Bologna, Corso d’Augusto 237, 47921 Rimini, Italy

**Keywords:** *Castanea sativa* Mill., Sweet chestnut, Cytotoxicity, Apoptosis, Chemoprevention, Flow cytometry, Jurkat cells

## Abstract

**Background:**

Chemoprevention represents the possibility to prevent, stop or reverse the cancerogenetic process. In this context the interest towards natural extracts and botanical drugs has constantly grown due to their phytochemical content. *Castanea sativa Mill.* (CSM) extracts showed to exert positive effect in the prevention/counteraction of chronic/degenerative diseases, therefore, we evaluated the potential chemopreventive effect of CSM bark extract.

**Methods:**

Flow cytometry (FCM) analyses of Jurkat cells treated with CSM bark extract (0–500 μg·mL^−1^) for 24–72 h allowed evaluating its cytotoxicity and ability to induce apoptosis through the intrinsic or extrinsic pathways. Moreover, to evaluate CSM bark extract selectivity towards cancer cells, its cytotoxic and pro-apoptotic effect was also evaluated in human peripheral blood lymphocytes (PBL).

**Results:**

CSM bark extract induced apoptosis in Jurkat cells in a dose- and time- dependent manner activating the extrinsic pathways as evidenced by the increase of activated caspase-8 positive cells. Moreover, IC_50_ calculated after 24 h treatment resulted 304 and 128 μg·mL^−1^ in PBL and Jurkat cells respectively.

**Conclusions:**

Our data suggest that CSM bark extract might be considered an interesting potential anti-cancer agent, since it induces apoptosis in cancer cells without appreciable cytotoxic effects on non-transformed cells.

**Electronic supplementary material:**

The online version of this article (doi:10.1186/s12906-017-1756-6) contains supplementary material, which is available to authorized users.

## Background

Cancer is a complex process comprised of at least three steps: initiation, an irreversible phase due to a DNA damage subsequent to exposure of normal cells to carcinogenic agents, promotion, a reversible step characterized by the clonal expansion of initiated cells that go beyond the normal mechanisms of cell proliferation and survival regulation, progression, an irreversible process in which additional genetic changes increase proliferative, invasive and metastatic potential of tumour cells [1].

Beside traditional therapeutic interventions such as chemotherapy, surgical removal and radiation therapy, chemoprevention has acquired great relevance in the fight against cancer. Chemoprevention through the use of synthetic or natural compounds, represents the possibility to inhibit, stop or reverse the process of carcinogenesis suppressing or preventing either the initial phases of carcinogenesis or delay the progression of premalignant cells to invasive disease [1, 2]. To prevent the initiation step, blocking agents can act inducting detoxification enzymes or blocking carcinogen formation, while suppressing agents counteract cancer promotion slowing cell division and inducing apoptosis and differentiation of neoplastic cells. Therefore, the inhibition of phase I and the induction of phase II drug metabolizing enzymes, the scavenging of free radicals/ultimate carcinogens, the induction of DNA repair constitute important anti-initiation chemopreventive actions, while the inhibition of clonal expansion by cell-cycle arrest, the induction of terminal differentiation, the modulation of signal transduction, inflammation, angiogenesis, immunomodulation, hormone modulation represent fundamental anti-promotion chemopreventive actions [1, 3–6]. Finally, the induction of apoptosis of initiated or neoplastic cells is one of the main mechanism to inhibit tumor growth [7]. Numerous studies demonstrated that alterations in cell death induction pathways are important for cancer development and are influencing the response to chemotherapy [8]. Moreover, a promising chemopreventive agent must show selectivity towards cancer cells and low toxicity on non-transformed cells [9, 10].

In the last decades, the interest towards natural extracts and botanical drugs has constantly grown. Plants have been used in traditional medicine all over the world for centuries and a great body of scientific literature is now reconsidering, the role of botanicals in new drugs development [11]. Plant extracts serve as sources of a plethora of bioactive molecules able to interact and affect different biochemical pathways. Many plant extracts, rich in bioactive compounds, are now emerging as key modulators of cancer risk and other chronic pathological conditions, such as cardiovascular and neurodegenerative diseases [6, 12–16].

*Castanea sativa Mill*. (CSM), better known as sweet chestnut, is a tree belonging to the Fagaceae family. It originates from the Mediterranean area, where chestnut had represented an important food source. Beside its importance as food, sweet chestnut has been used for centuries in folk medicine to face a wide array of disorders such as cold, diarrhoea asthma and bronchitis. Sweet chestnut leaves infusion was used to treat cough, in the middle age it was used against heart disorders and in the treatment of back pain and rheumatism. The bark, due to its high tannin content, was used for its astringent properties to stop bleeding [17].

In the last decade, some studies investigated sweet chestnut extracts composition and described CSM as a source of phenolic compounds such as tannins, lignan constituents and antioxidant compounds [18, 19]. Using Vero cells as a model system, Lupini et al. [20] evaluated the antiviral activity of Chestnut wood extracts used in animal feed preparation and containing hydrolysable tannins, against avian reovirus and avian metapneumovirus. Frankic et al. [21] demonstrated that a commercially available CSM wood extract containing 73% tannins, added to animal feed, reduced oxidative stress biomarkers such as urine isoprostanes and prevented lymphocytes DNA damage in young pig exposed to n-3 PUFA-induced oxidative stress*.*

Almeida et al. [22] recently described the photoprotective effect of CSM leaf extract against UV irradiation (UVA 0.5 J/cm^2^) in human keratinocyte cell line (HaCaT). Their study suggested that CSM leaf extract exerted both a direct antioxidant effect, by scavenging ^1^O_2_ and a protective action against DNA damage, as shown by the reduction of micronuclei frequency in CSM leaf extract treated cells.

We recently characterized by HPLC-DAD-MS analysis the phenolic composition of a CSM bark extract showing that it is rich in tannins and phenolic compounds such as: castalin, vescalin, castalgin, vescalgin, ellagic and gallic acids and demonstrated its antioxidant and cytoprotective effects in cultured cardiomyocytes exposed to H_2_O_2_ induced oxidative stress [23]. Ellagic and gallic acids as well as tannins have been demonstrated to induce apoptosis in different in vivo and in vitro models [24–28].

### Aim of the study

Aim of this study was to evaluate CSM bark extract as a candidate chemopreventive agent. In particular antiproliferative and pro-apoptotic effects were analysed in human T leukemia cells (Jurkat cells), a commonly used cell line in the study of susceptibility of cancer cells to drugs and natural bioactives [9, 29, 30]. Moreover, possible molecular mechanisms were analysed. Studies were focused on alteration of mitochondrial transmembrane potential and on modulation of caspase-8 to understand if the induction of apoptosis was triggered by the intrinsic or the extrinsic pathway. The level of p53 and Bax, two proteins involved in development and progression of cancer were also analysed. The dysregulation of tumour suppressor gene p53 and the dysfunction of pro-apoptotic protein Bax can lead to uncontrolled growth and carcinogenesis. Conversely, the up-regulation of their levels can be exploited as chemopreventive mechanism [31, 32]. Finally, the possible selectivity of CSM bark extract was evaluated by testing its activity on non-transformed human peripheral blood lymphocytes (PBL).

## Methods

### Materials

Ethanol, Fetal Bovine Serum (FBS), Formaldehyde, Histopaque-1077, L–Glutamine (L-GLU), Methanol, 3-(4,5-dimethylthiazol-2-yl)-2,5-diphenyltetrazolium (MTT), Penicillin-Streptomycin solution (PS), Phosphate Buffered Saline (PBS), Phytohemagglutinin (PHA), Roswell Park Memorial Institute (RPMI) 1640 medium were purchased from Sigma-Aldrich, St Louis, MO. Guava Caspase Reagent, Guava Cell-Cycle Reagent, Guava Mitopotential Reagent, Guava Nexin Reagent, Guava ViaCount Reagent were purchased from Merck Millipore, Darmstadt, Germany. Purified mouse anti-Bax, PE conjugated p53 antibody were purchased from BD Bioscences, San Jose, California, USA. Anti-Mouse IgG Secondary Antibody FITC conjugate was purchased from Thermo Fisher, Waltham, Massachusetts, USA.

### CSM bark extract

CSM bark extract supplied by SilvaTeam (San Michele di Mondovì, Italy) was obtained by low pressure heating treatment as previously described [33]. It appears as a brown powder, which is preserved at room temperature and protected from light. We previously characterized, by HPLC-DAD-MS analysis, the same batch of CSM bark extract used in this manuscript demonstrating that it is rich in phenolic compounds such as: castalin, vescalin, castalgin, vescalgin, ellagic acid and gallic acid [23]. A 10 mg/mL working solution, used in further experiments was obtained by solubilising the powder in RPMI with 20% *v/v* DMSO. DMSO concentration was always in the range 0.05–1% in all the experimental conditions.

### Cell cultures

Jurkat cells were grown at 37 °C and 5% CO_2_ in RPMI-1640 supplemented with 1% PS, 10% FBS and 1% L-Glutammine. To maintain exponential growth, the cultures were divided every third day in fresh medium. The cell density did not exceed the critical value of 3 × 10^6^ cell·mL^−1^ of medium.

PBL were isolated by density gradient centrifugation with Histopaque-1077 from whole peripheral blood of 5 donors AVIS (Italian Association of voluntary Blood donors). The donors had the following characteristics: under the age of 35, healthy, non-smoker and with non-known exposure to genotoxic chemicals or radiation. PBL were cultured at 37 °C and 5% CO_2_ in RPMI-1640 supplemented with 1% PS, 15% FBS, 1% L-Glutammine, and 0.5% phytohemagglutinin (PHA).

### Treatments

Jurkat cells were seeded at the density of 3.25 × 10^5^ cells/ml in complete medium and treated for 24, 48 or 72 h with 0, 25, 50, 100, 250, 500 μg·mL^−1^ of CSM bark extract and incubated at 37 °C and 5% CO_2_.

PBL were seeded at the density of 4 × 10^5^ cells/ml and cultured for 44 h in the presence of PHA and then treated with 0, 25, 50, 100, 250, 500 μg·mL^−1^ concentration of CSM bark extract and incubated at 37 °C and 5% CO_2_ for 24 h.

### Cytotoxicity by MTT assay

MTT assay was performed as previously described [34] Cells were incubated in 96-well flat-bottomed plates with 0.5 mg·mL^−1^ MTT for 4 h at 37 °C. At the end of the incubation, blue-violet formazan salt crystals were formed and dissolved by adding the solubilization solution (10% SDS, 0.01 M HCl), then the plates were incubated overnight in humidified atmosphere (37 °C, 5% CO_2_) to ensure complete lysis. The absorbance at 570 nm was measured using a multiwell plate reader (Wallac Victor^2^, PerkinElmer).

### Flow cytometry (FCM)

All FCM analyses were performed using a flow cytometer Guava easyCyte 5HT equipped with a class IIIb laser operating at 488 nm (Merk Millipore, Darmstadt, Germany).

### Cytotoxicity by FCM

The percentage of viable cells was assessed by FCM and analyzed with Guava ViaCount software. After 24, 48 and 72 h Guava ViaCount Reagent was added to the cells to discriminate viable and dead cells; the reagent contains the dye Propidium Iodide (PI) able to penetrate only the altered membrane of necrotic cells, bind covalently to DNA and emit red fluorescence. In contrast, cells with integral membrane are not permeable to PI, and then emit low red fluorescence. The obtained results were expressed as the percentage of live cells in treated cultures compared to that present in the control cultures.

### Apoptosis by FCM

The percentage of apoptotic cells was assessed by FCM and analyzed with Guava Nexin software. After 24, 48 and 72 h Guava Nexin Reagent was added to the cells, the reagent contains two dyes, 7-aminoactinomycin D (7-AAD) and Annexin-V-PE. As previously described for PI, 7-AAD allows the discrimination between live and dead cells, while Annexin-V-PE allows identification of apoptotic cells by binding to phosphatidylserine and emitting yellow fluorescence. In particular, live cells are 7-AAD and Annexin-V-PE negative, apoptotic cells are 7-AAD negative and Annexin-V-PE positive and necrotic cells are 7-AAD and Annexin-V-PE positive. The obtained results were expressed as the percentage of apoptotic cells in treated cultures compared to those present in the control cultures.

### Cellc-cycle by FCM

The percentage of cells in the different stages of cell-cycle was assessed by FCM and analyzed, after 24, 48 and 72 h treatment, with Guava Cell-Cycle software. Cells were fixed and permeabilized with ice-cold 70% ethanol and washed with PBS. Then, cultures were resuspended in Guava Cell-Cycle Reagent that contains the dye PI. The PI is so able to penetrate the membrane of cells, bind covalently to DNA and emit red fluorescence. In particular, cells which initially are in G_0_/G_1_ phase begins to synthesize DNA in S phase, until the complete duplication in G_2_/M. Therefore, the cells in G_2_/M phase have a double fluorescence compared to those in G_0_/G_1_ phase, while the cells in S phase have an intermediate fluorescence.

The obtained results were expressed as the percentage of cells in the different stages of the cell-cycle in treated cultures compared to that present in the control cultures.

### Extrinsic apoptosis pathway by FMC

The percentage of cells with caspase-8 activated was assessed by FCM and analyzed, after 72 h treatment, with Guava Caspase software as previously reported [31]. Guava Caspase-8 Reagent was added to the cells, the reagent contains two dyes, FLICA, an inhibitor of caspase-8, conjugated FAM and 7-AAD. As previously described, 7-AAD allows to discriminate between live and dead cells, while FLICA is cell permeable. Once inside the cell, FLICA binds covalently to the activated caspase-8 and emit green fluorescence. In particular, live cells result 7-AAD and FLICA negative, cells with activated caspase-8 are 7-AAD negative and FLICA positive and necrotic cells are 7-AAD and FLICA positive. The obtained results were expressed as the percentage of cells with activated caspase-8 in treated cultures compared to that present in the control cultures.

### Intrinsic apoptosis pathway by FCM

The percentage of apoptotic cells with altered mitochondrial membrane potential was assessed by FCM and analyzed, after 24, 48 and 72 h treatment, with Guava Mitopotential software. Cells were stained with the Guava Mitopotential Reagent that contains two dyes, JC-1 and 7-AAD. 7-AAD allows to discriminate between live and dead cells, as previously described, while JC-1 is a permeant cationic dye that fluoresces either green and orange depending upon mitochondrial membrane potential. In particular, live cells (polarized cells) are 7-AAD negative and orange JC-1 positive, apoptotic cells (depolarized cells) are 7-AAD negative and green JC-1 positive, and necrotic cells are 7-AAD positive and green JC-1 positive. The obtained results were expressed as the percentage of apoptotic cells with altered mitochondrial membrane potential in treated cultures compared to that present in the control cultures.

### Protein levels by FCM

The mean fluorescence intensity value of p53 and Bax proteins was analyzed, after 24, 48 and 72 h treatment, by FCM with Guava Incyte software. Cells were fixed in PBS plus formaldehyde 4% and permeabilized in 90% cold methanol. Cells were then incubated with Anti-p53-PE antibody and washed or with Anti-Bax primary antibody, washed, incubated with fluorescein isothiocyanate-labeled secondary antibody and then analyzed. The obtained results were expressed as mean fluorescence intensity value of cells in treated cultures compared to that present in the control cultures. Non-specific binding was excluded by isotype control.

### Statistical analysis

All results are expressed as mean ± standard error mean (SEM) of at least five independent experiments. For statistical analysis of apoptosis and cell-cycle we used the Analysis of Variance for paired data (repeated ANOVA), followed by Bonferroni as post-test. For statistical analyses of apoptosis pathways and protein levels we used the t-test for paired data. All the statistical analyses were performed using Prism Software 6.

## Results

**Fig. 1:**
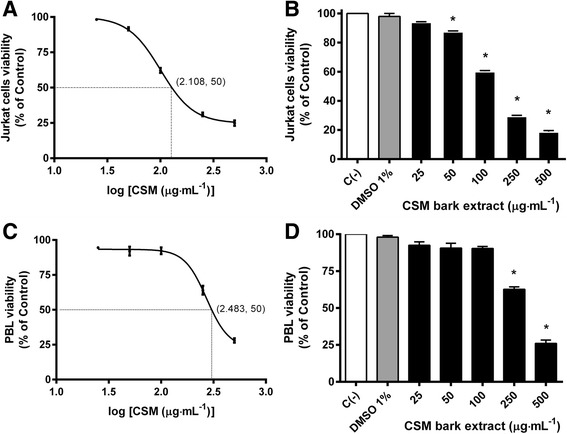
Jurkat cells and PBL viability after 24 h treatment with CSM bark extract Cell viability was evaluated by FCM (panels **a** and **c**) and MTT assay (panels **b** and **d**) as described in Methods. IC_50_ was obtained by curve fitting of viability after 24 h treatment with CSM bark extract for Jurkat cells (**a**) and PBL (**c**). Data are presented as mean ± SEM of five independent experiments. Data were analyzed by repeated ANOVA followed by Bonferroni post-test. *p < 0.05 vs control.

Cytotoxicity on Jurkat cells was evaluated by FCM (Fig. 1a) and confirmed by MTT assay (Fig. 1b), after 24 h treatment with 0–500 μg·mL^−1^ CSM bark extract concentrations. The IC_50_ value, obtained by curve fitting, was 128 μg·mL^−1^ for Jurkat cells.

In order to verify the selectivity against tumour cells, PBL were treated for 24 h with 0–500 μg·mL^−1^ CSM bark extract. The effect of CSM bark extract on PBL viability was evaluated by FCM (Fig. 1c) and by MTT assay (Fig. 1d). Cell viability remained above 60% up to the 250 μg·mL^−1^ concentration. In fact, the IC_50_ value, obtained by curve fitting was 304 μg·mL^−1^, 2.4 times higher than that obtained on Jurkat cells.

**Fig. 2:**
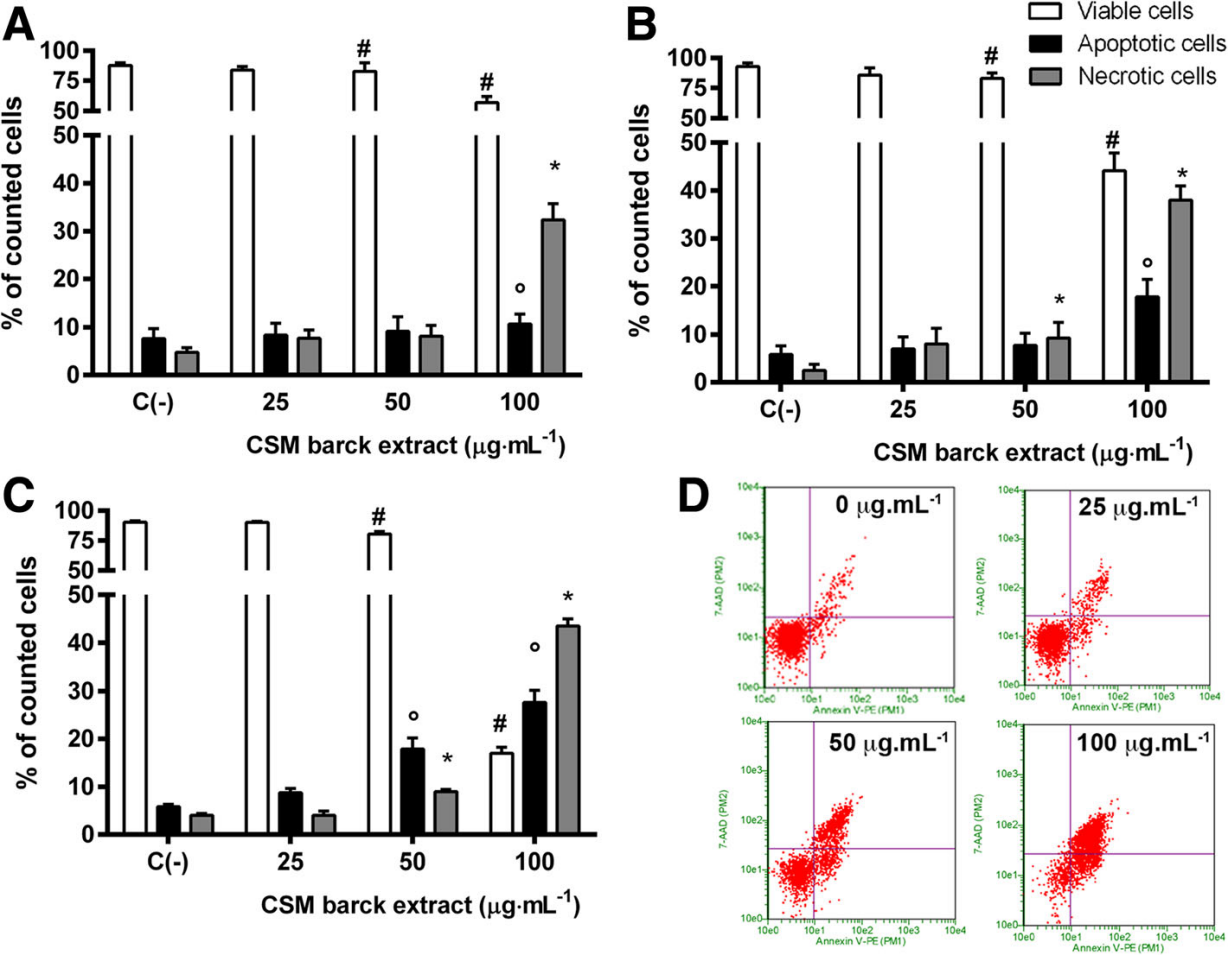
Fraction of viable, apoptotic and necrotic Jurkat cells treated with CSM bark extract for 24 h (**a**), 48 h (**b**), 72 h (**c**) and representative dot plot of apoptosis analysis at 72 h treatment (**d**). Apoptosis was evaluated by FCM as described in Methods. Each bar represents the mean ± SEM of five independent experiments. Data were analyzed by repeated ANOVA followed by Bonferroni post-test. # *p* < 0.05 vs control viable cells, ° *p* < 0.05 vs control apoptotic cells, *p < 0.05 vs control necrotic cells

**Fig. 3:**
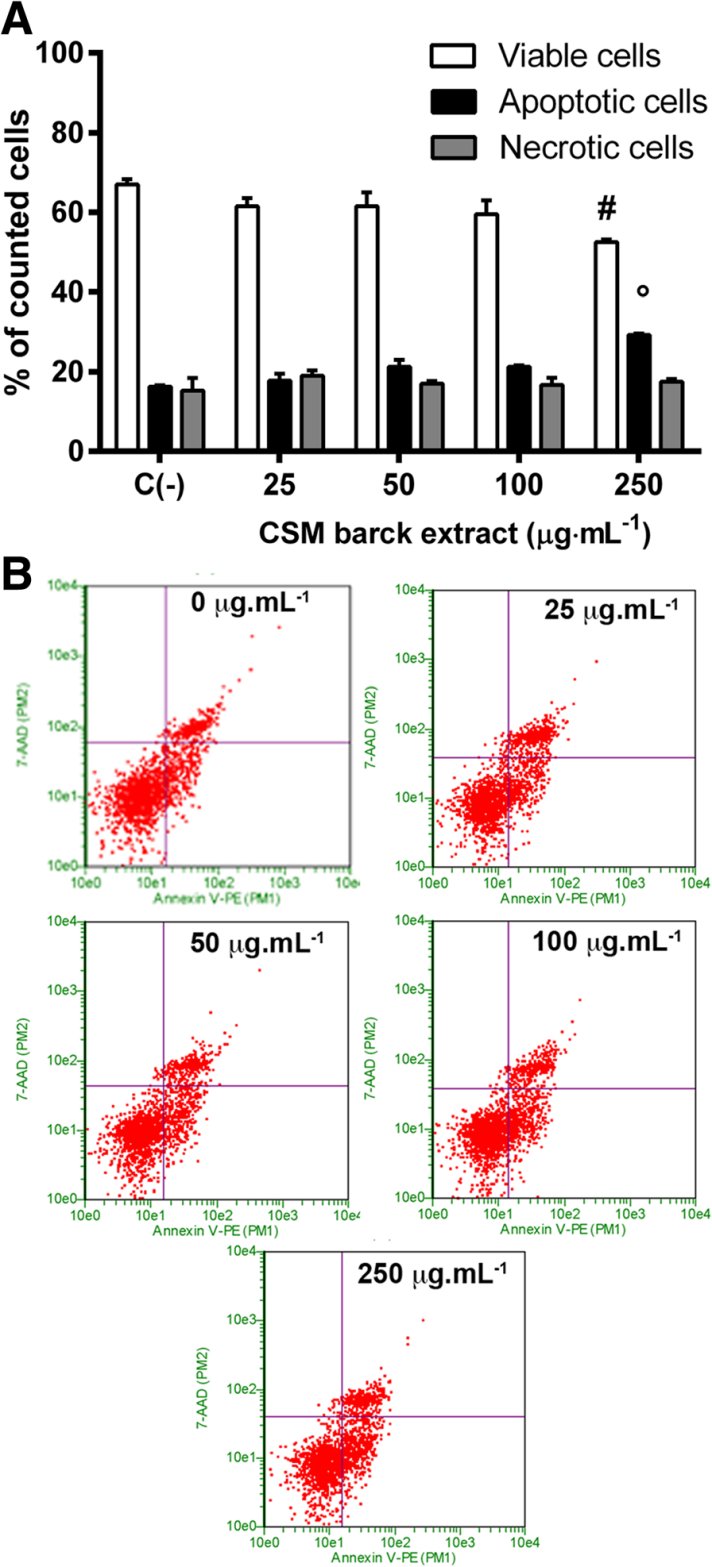
Fraction of viable, apoptotic and necrotic PBL treated with CSM bark extract at the indicated concentrations for 24 h (**a**) and representative dot plot of apoptosis analysis at (**b**). Apoptosis was evaluated by FCM as described in Methods. Each bar represents the mean ± SEM of five independent experiments. Data were analyzed by repeated ANOVA followed by Bonferroni post-test. #*p* < 0.05 vs control viable cells, ° *p* < 0.05 vs control apoptotic cells

In order to assess the involvement of a specific cell death mechanism responsible for the cytotoxicity, the induction of apoptosis was investigated in both Jurkat cells and PBL (Figs. 2 and 3 respectively).

In Jurkat cells CSM bark extract induced apoptosis in a dose- and time- dependent manner, as shown in Fig. 2. The double staining Annexin V-PE / 7-AAD revealed, after 24 h treatment, a statistically significant increase in apoptotic cells at the concentration of 100 μg·mL^−1^ (10.7 ± 0.7% vs 7.6 ± 0.7% in the control) (Fig. 2a).

After 48 h, 100 μg·mL^−1^ extract caused a greater increase in apoptotic cells compared to 24 h. Apoptotic cells were 3 times higher than in controls (17.9 ± 1.2% vs 5.8 ± 0.6%) (Fig. 2b).

Apoptosis increased up to 5 times after 72 h respect to control (27.6 ± 2.5% vs 5.8 ± 0.5%) and a statistically significant increase in apoptotic fraction was also measured at 50 μg·mL^−1^ (17.9 ± 2.3% vs 5.8 ± 0.5%) (Fig. 2c). A strong increase in necrotic cells was also evident at 100 μg·mL^−1^ respect to control. Representative dot plots of apoptosis analysis at 72 h treatment are shown in Fig. 2d. To further confirm the CSM bark extract apoptotic effect, nuclear condensation and fragmentation were evaluated by fluorescence microscopy as shown in Additional file 1.

To assess the involvement of a specific cell death mechanism responsible for the extract cytotoxicity on PBL, the induction of apoptosis was investigated after 24 h treatment with CSM bark extract concentrations less then IC_50_ (0–250 μg·mL^−1^).

A statistically significant increase in apoptotic cells was detected only at 250 μg·mL^−1^ (29 ± 0.3% vs 16 ± 1.3% in the controls) (Fig. 3a), Representative dot plots of apoptosis analysis at 24 h treatment are shown in Fig. 3b.

In order to evaluate whether the induction of apoptosis caused by CSM bark extract was an independent or subsequent event to cell-cycle arrest, the Jurkat cells were treated with 25, 50, 100 μg·mL^−1^ CSM bark extract concentrations for 24 h, 48 h, or 72 h.

**Fig. 4:**
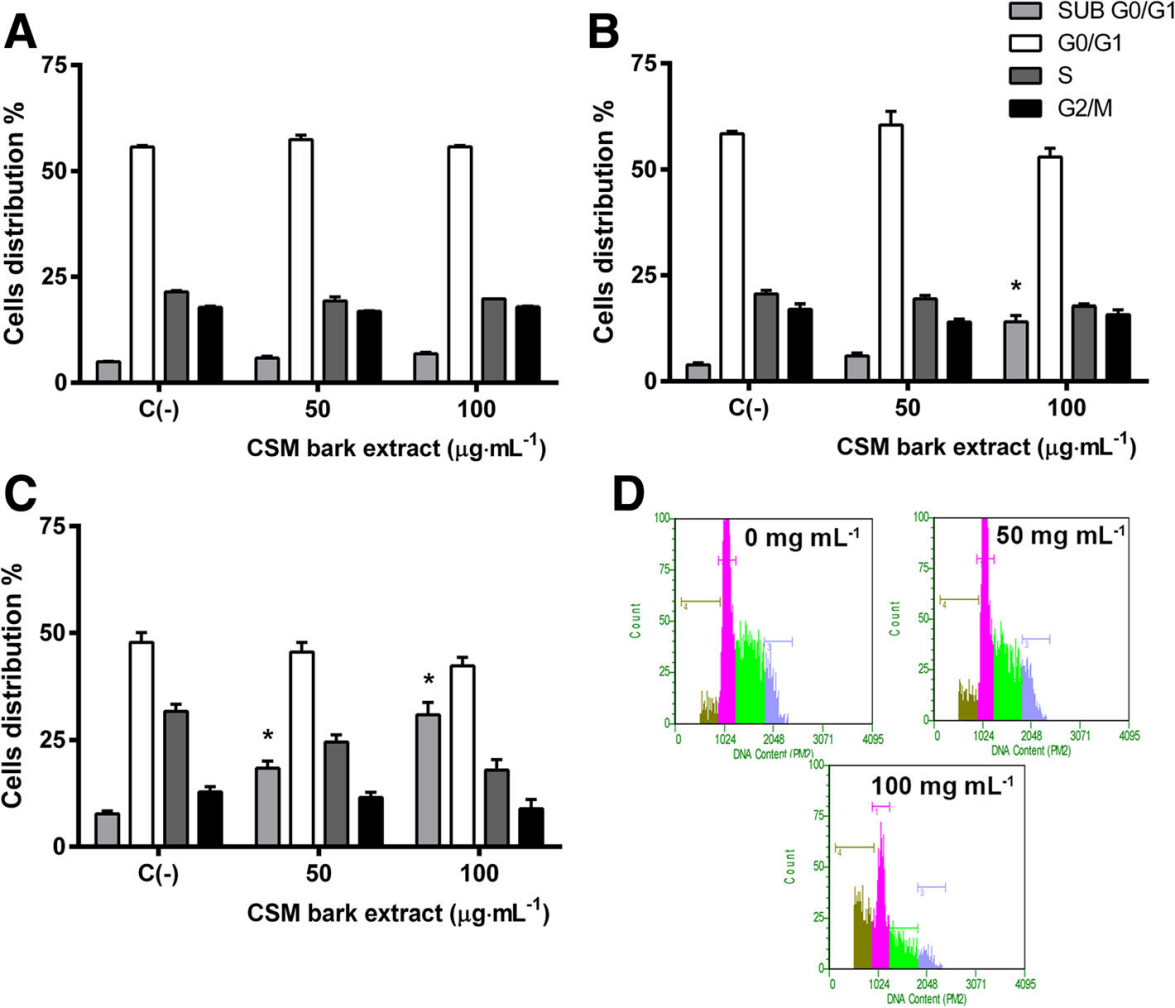
Effect of CSM bark extract treatment on cell-cycle. Jurkat cells were treated with extract for 24 h (**a**), 48 h (**b**), 72 h (**c**) and representative histograms of cell-cycle analysis at 72 h treatment (**d**). Cellular distribution in the different phases was evaluated by FCM as described in Methods. Each bar represents the mean ± SEM of five independent experiments. Data were analyzed by repeated ANOVA followed by Bonferroni post-test. * *p* < 0.05 vs control sub G_0_/G_1_

PI staining highlighted the percentage of cellular distribution in the different phases of the cell-cycle. At 24, 48 and 72 h treatment and at each tested concentration no cytostatic effect was evidenced (Fig. 4 a, b, c). Sub G_0_/G_1_ population reflected the apoptosis induced by CSM bark extract and no cell-cycle arrest was observed in any specific phase. Representative histograms of cell-cycle analysis at 72 h treatment are shown in Fig. 4d.

Next, to evaluate whether the induction of apoptosis caused by CSM bark extract was triggered by the activation of the extrinsic or the intrinsic pathway, Jurkat cells were treated for 72 h with 50 μg·mL^−1^ extract corresponding to the time and the concentration at which apoptosis was 3 times higher than in controls, but necrosis was still negligible (8% vs 4% in the control). The 100 μg·mL^−1^ concentration was not employed, nevertheless even if it induces apoptosis, since it also strongly increases necrosis (43.5% vs 4% in the control).

**Fig. 5:**
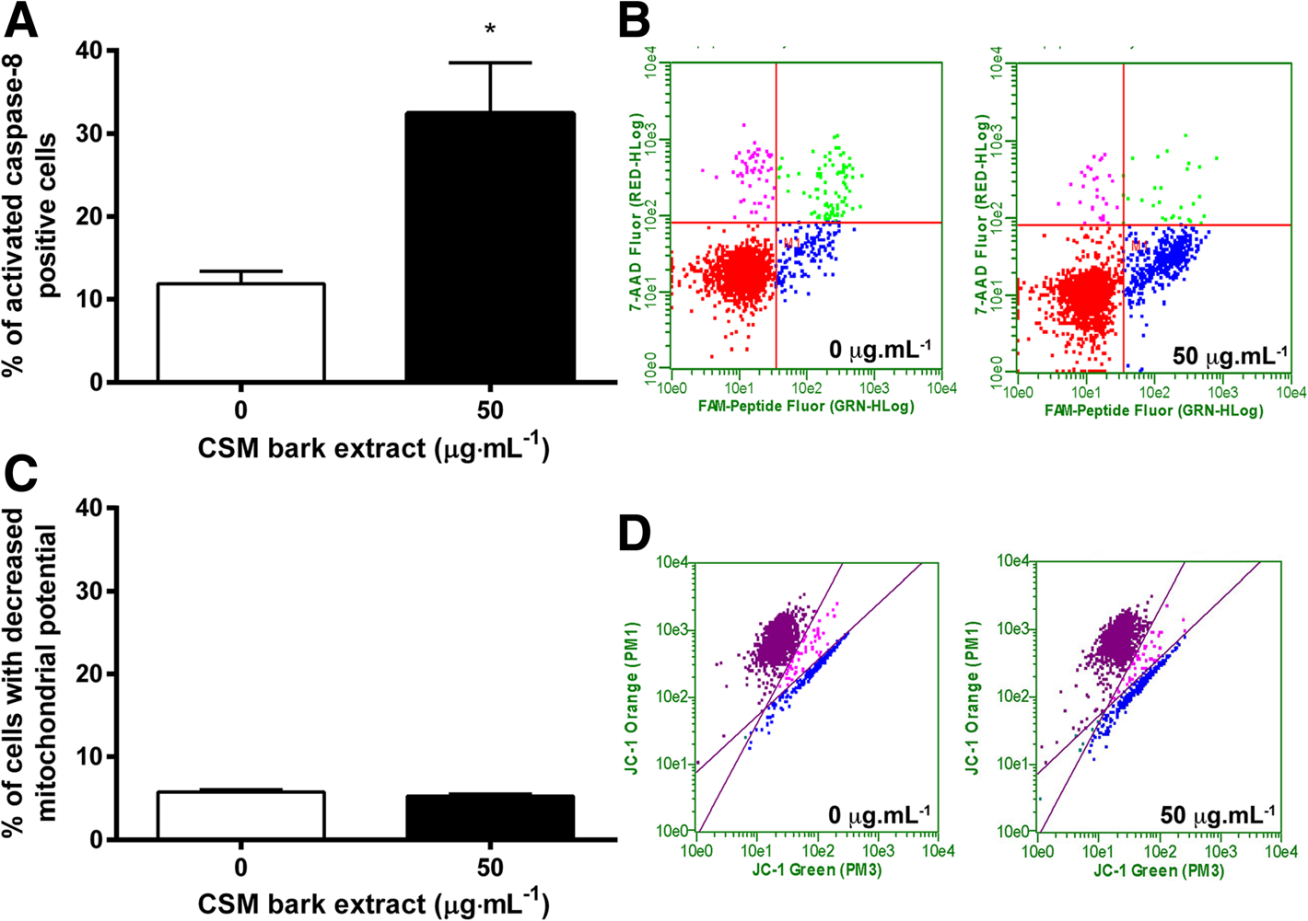
Fraction of apoptotic cells with active caspase-8 (**a**) and with altered mitochondrial membrane potential (**c**) after 72 h treatment with 50 μg·mL^−1^ CSM bark extract. Panels **b** and **d** show representative dot plot of cells with active caspase-8 and altered mitochondrial membrane potential respectively. Active caspase-8 and mitochondrial membrane potential were evaluated by FCM as described in Methods. Each bar represents the mean ± SEM of five independent experiments. Data were analyzed by t-test for paired data. *p < 0.05 vs control

Figure 5a represents the percentage of cells with activated caspase-8, measured by FCM. Data suggest that CSM bark extract induced apoptosis occurred by the activation of the extrinsic apoptotic pathway, while the intrinsic apoptotic pathway appeared not to be involved. A three times increase in apoptotic cells was evidenced in both activated caspase-8 assay (32.4 vs 11.8%) and the annexin-V assay (17.8 vs 5.9%) following 50 μg·mL^−1^ treatment. Conversely, cells characterized by JC-1 green fluorescence did not increase with respect to controls (Fig. 5c). Representative dot plots of activated caspase-8 and altered mitochondrial potential analysis are shown in Fig. 5b and d respectively.

Since the pro-apoptotic effect of CSM bark extract is particularly marked after 72 h treatment at 50 μg·mL^−1^ extract concentration, p53 and Bax protein levels were analyzed under these experimental conditions.

**Fig. 6:**
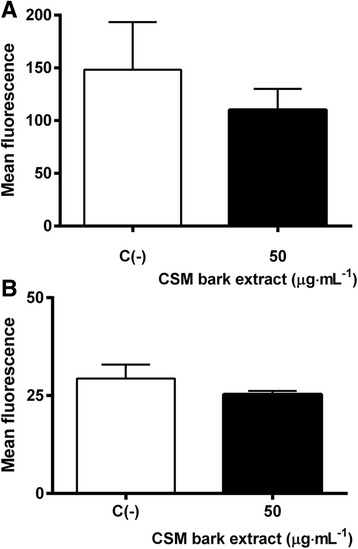
p53 (**a**) and Bax (**b**) mean fluorescence intensity after 72 h treatment with 50 μg·mL^−1^ CSM bark extract at the indicated concentration. p53 and Bax levels were evaluated by FCM as described in Methods. Each bar represents the mean ± SEM of five independent experiments. Data were analyzed by t-test for paired data

As shown in Fig. 6, p53 (Fig. 6a) and Bax (Fig. 6b) protein levels, measured by FCM, were unchanged, as compared with controls.

To exclude that the loss of mitochondrial potential and the increase in p53 and Bax levels might occur before 72 h treatment, their levels were investigated also at 24 and 48 h, revealing that these parameters were not affected by CSM bark extract treatment at any time data not shown.

## Discussion

The aim of this study was to evaluate if CSM bark extract elicited chemopreventive activity in Jurkat cells.

Since a large number of natural compounds or plant extracts have been suggested as potential modifiers of numerous chronic/degenerative diseases [35–38], an increasing number of studies are now exploring the field of plant phytochemicals with the aim to identify chemopreventive agents as modulators of the different stages of carcinogenesis [39, 40].

Even though CSM wood, bark and leaf extracts have recently attracted interest due to their photoprotective, neuroprotective, cardioprotective and antioxidant properties that suggest their suitability for the prevention of chronic and degenerative diseases [22, 23, 41], to our knowledge no study has previously evaluated the pro-apoptotic effect of CSM extracts on cancer cells. In this study, we evaluated the effects of CSM bark extract in human T leukemia cells. The specific mechanism of cell death (apoptosis and/or necrosis) and the ability to modulate the cell-cycle were investigated, in order to assess whether cytotoxic and cytostatic effects were independent or subsequent events. Results suggest that the CSM bark extract is able to induce apoptosis in Jurkat cells in a dose- and time- dependent manner. However, after 72 h treatment at 100 μg·mL^−1^ a strong increase (up to 11 times) of necrosis was also recorded with respect to the control.

Conversely, CSM bark extract did not exhibit antiproliferative effect, as evidenced by the distribution of cells in the different phases of the cell-cycle, which was not affected at all times and concentrations tested.

Altogether, these findings suggest that the induction of apoptosis in Jurkat cells is triggered by CSM bark extract without any cell-cycle modulation.

To identify the molecular pathways involved in CSM ability to trigger apoptosis, we analyzed caspase-8 levels and the mitochondrial membrane potential. Caspase-8 triggers the extrinsic pathway inducing the binding of signaling molecules with their own specific receptors on the plasmatic membrane, such as Fas-L to Fas receptor, belonging to the superfamily of TNF-NGF receptors [42].

The opening of pores on the mitochondrial membrane causes permeability transition phenomena, with consequent lowering of the electric potential difference and induction of apoptosis by the intrinsic pathway [43].

After 72 h treatment at 50 μg·mL^−1^, the significant increase in apoptotic cells (Annexin V-PE positive / 7-AAD negative) perfectly matched with the increase in activated caspase-8 in Jurkat cells. These data suggest that the pro-apoptotic effect of CSM bark extract was due to the involvement of the extrinsic pathway, since apoptosis did not correlate with a loss of mitochondrial transmembrane potential. The hypothesis was corroborated by FCM analysis, which showed that Bax levels were not influenced by CSM bark extract treatment. Bax is a pro-apoptotic protein. Its activation contributes to an increase of cytosolic calcium levels actively transported into the mitochondria with consequent alteration of the membrane potential and formation of pores that increase permeability and result in the release of pro-apoptotic factors, such as cytochrome c. Cytochrome c binds Apaf1 and generates a protein complex called apoptosome, which activates the pro-caspase-9 inducing apoptosis [44].

The lack of Bax level increase is in agreement with data on mitochondrial potential, and suggest that the CSM bark extract pro-apoptotic capacity was probably due to the activation of the extrinsic pathway.

**Fig. 7:**
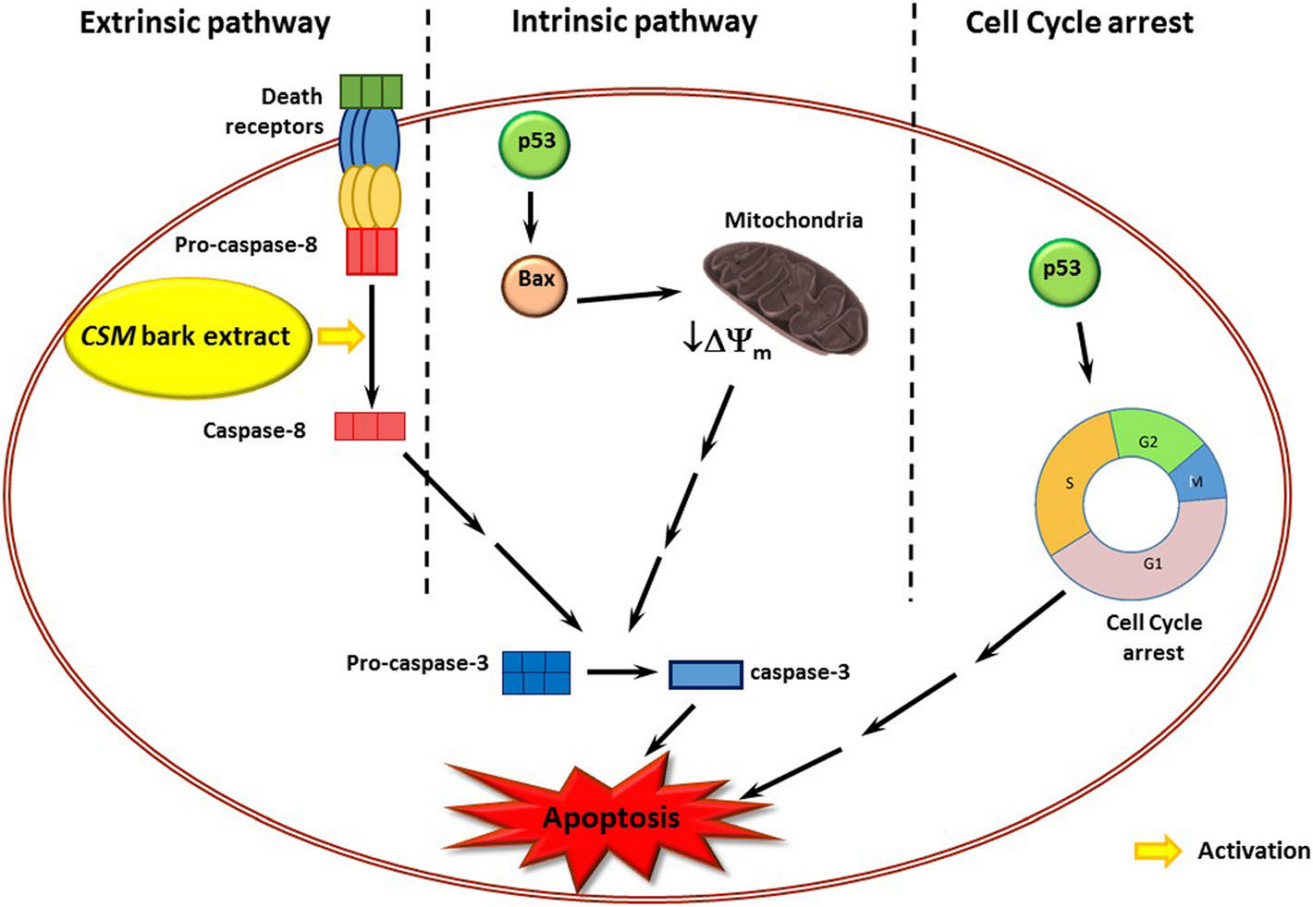
Schematic mechanism of anticancer activity of CSM bark extract

When the DNA is damaged, p53, known as “the guardian of the genome”, is able to slow down cell-cycle, to allow the repair systems to act, or, when the damage is irreparable, to induce apoptosis to selectively eliminate aberrant cells [45]. In this context, it is interesting to note that cancer cells are generally characterized by the absence of p53 or the presence of mutated p53, so they are no longer responsive to its control mechanisms [46]. Moreover, most of the conventional anticancer agents require the presence of intact p53 to exert their pharmacological activity [47]. Jurkat cells present a mutated p53, [48] therefore CSM bark extract-induced cell death, although characterized by molecular apoptotic marker, support the hypothesis of a p53-independent mechanism. The proposed pro-apoptotic mechanism of CSM bark extract is summarized in the scheme reported in Fig. 7.

Essential features of a good chemopreventive agent are selectivity toward cancer cells and low toxicity towards non-transformed cells [49]. Results obtained with CSM on PBL from healthy donors, the non-transformed counterparts of Jurkat cells, suggest that the extract might be considered a partially selective cytotoxic agent, due to its ability to induce higher cell death in transformed cells. In fact, IC_50_ for PBL was more than twice that of Jurkat cells (304 vs 128 μg·mL^−1^) and, in PBL, the induction of apoptosis by CSM bark extract was revealed after 24 h only at the highest tested dose.

Most tumor cells exhibit alterations in the ability to mature into adult non-proliferating cells, conserving therefore, a high proliferative state. Since the induction of terminal differentiation generates cells with no or limited replicative capacity that enter more easily apoptosis [50], an interesting challenge could be to investigate the CSM bark extract potential to stimulate differentiation. Studies are in progress to address this possibility.

## Conclusions

In conclusion, our paper casts just a first glance into the potential chemopreventive activity of CSM bark extract on tumor cells. Our data warrant further scrutiny to deepen CSM extracts chemopreventive potential, to confirm its proapoptotic mechanism through the investigation of different endpoints such as caspases 3, 9, 12 and other proteins involved in apoptosis and to investigate which components are responsible for its effects.

## Additional file

Additional file 1: Morphological analysis of Jurkat cells in the absence and presence of CSM bark extract. Apoptosis-associated nuclear condensation and fragmentation were evaluated in untreated (A) and treated (B), (50 μg·mL^−1^ CSM bark extract for 72 h) Jurkat cells by fluorescence microscopy at 100× magnification. 3 × 10^5^ Jurkat cells were loaded into cytospin chambers and centrifuged ad 450 rpm for 10 min. Cells were then fixed in formaldehyde 3.7%, permeabilised in 0.15% triton X-100 and nuclei were stained with 0.5 μM Hoechst 33,258 as reported by Henry et al. [51]. White arrows indicate condensed and/or fragmented nuclei as a marker of apoptosis. (TIFF 4797 kb)

## Abbreviations

7-AAD: 7-amminoactinomycin
CSM: *Castanea sativa* Mill.
FAM: Fluorescein
FCM: Flow cytometry
FLICA: Fluorocrome-labeled inhibitors of caspases
JC-1: 5,5′,6,6′-tetrachloro-1,1′,3,3′tetraethylbenzimidazolcarbocyanine iodide
L-GLU: L-Glutamine
PBL: Human perpheral blood lymphocytes
PE: Phycoerythrin
PHA: Phytohemagglutinin
PI: Propidium iodide
PS: Penicillin-streptomycin solutio
RPMI: Roswell Park Memorial Institute 1640 medium
