# Supplementary figures and images for: Castanea sativa Mill. bark extract exhibits chemopreventive properties triggering extrinsic apoptotic pathway in Jurkat cells
Source: BMC Complement Altern Med. 2017 May 5;17:251. doi: 10.1186/s12906-017-1756-6 (PMC5420104; doi:10.1186/s12906-017-1756-6)

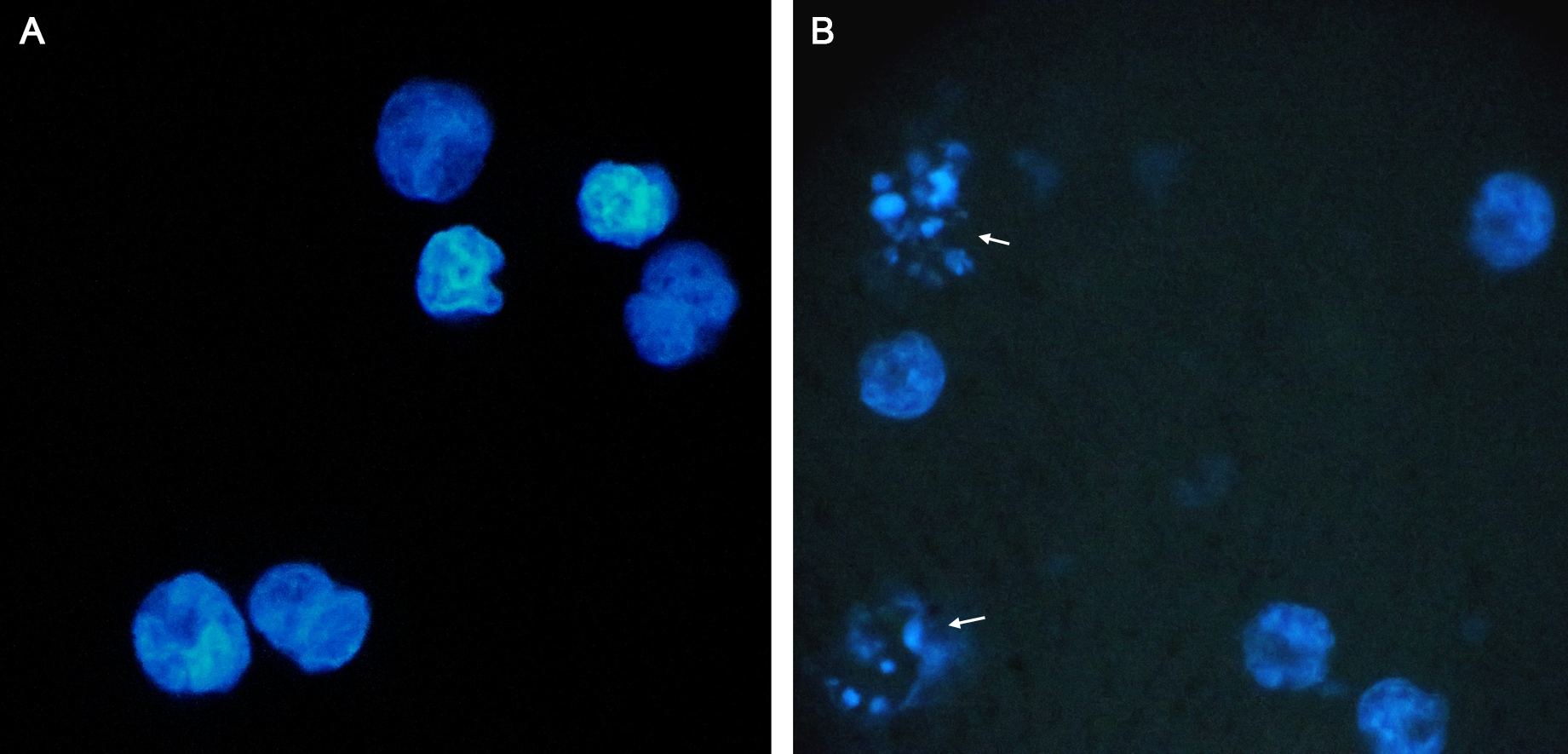

Supplement: Additional file 1: — Morphological analysis of Jurkat cells in the absence and presence of CSM bark extract. Apoptosis-associated nuclear condensation and fragmentation were evaluated in untreated (A) and treated (B), (50 μg·mL−1 CSM bark extract for 72 h) Jurkat cells by fluorescence microscopy at 100× magnification. 3 × 10^5^ Jurkat cells were loaded into cytospin chambers and centrifuged ad 450 rpm for 10 min. Cells were then fixed in formaldehyde 3.7%, permeabilised in 0.15% triton X-100 and nuclei were stained with 0.5 μM Hoechst 33,258 as reported by Henry et al. [51]. White arrows indicate condensed and/or fragmented nuclei as a marker of apoptosis. (TIFF 4797 kb) [file 12906_2017_1756_MOESM1_ESM.tif]
